# The Body as a Tool for Anger Awareness—Differential Effects of Angry Facial and Bodily Expressions on Suppression from Awareness

**DOI:** 10.1371/journal.pone.0139768

**Published:** 2015-10-15

**Authors:** Minye Zhan, Ruud Hortensius, Beatrice de Gelder

**Affiliations:** 1 Brain and Emotion Laboratory, Department of Cognitive Neuroscience, Faculty of Psychology and Neuroscience, Maastricht University, The Netherlands; 2 Cognitive and Affective Neuroscience Laboratory, Tilburg University, Tilburg, The Netherlands; University of Montreal, CANADA

## Abstract

Emotional signals are perceived whether or not we are aware of it. The evidence so far mostly came from studies with facial expressions. Here, we investigated whether the pattern of non-conscious face expression perception is found for whole body expressions. Continuous flash suppression (CFS) was used to measure the time for neutral, fearful, and angry facial or bodily expressions to break from suppression. We observed different suppression time patterns for emotions depending on whether the stimuli were faces or bodies. The suppression time for anger was shortest for bodily expressions, but longest for the facial expressions. This pattern indicates different processing and detection mechanisms for faces and bodies outside awareness, and suggests that awareness mechanisms associated with dorsal structures might play a role in becoming conscious of angry bodily expressions.

## Introduction

In the course of daily interaction, people naturally display facial and bodily expressions that are easily recognized by others. Many studies have provided evidence that facial expressions can also be processed when they are not consciously perceived. For example, masked fearful faces orient spatial attention [1], influence the perception of simultaneously presented unmasked expression [2], elicit amygdala activation [3] and boost the connectivity between the amygdala and the colliculo-pulvinar pathway [4].

Traditionally, studies investigating non-conscious perception used visual masking techniques, as in the aforementioned studies. However, a recently developed paradigm called continuous flash suppression (CFS) [5], a variant of binocular rivalry, is increasingly used to investigate the processing of emotions outside visual awareness. In this paradigm, one eye is presented with the experimental stimulus, while the other eye is presented with a flashing, colorful “Mondrian” pattern, which renders the stimulus invisible for the participant. This offers stronger suppression than masking, and creates a more stable non-conscious perception for participants [6]. CFS exploits the rivalry of the dichoptic stimuli, hypothetically occurring at both monocular neurons in lower-level visual areas and binocular pattern selective neurons in higher visual areas [7]. Previous studies using this paradigm have shown that facial stimuli with certain emotional contents can be processed without awareness [8], and that these emotional contents can affect judgments made by participants afterwards [9].

Although CFS can generally suppress visual stimuli for a relatively long time, the actual duration of suppression tends to vary with the stimulus category as well as with certain properties of the stimuli. In particular, stimuli that are salient or meaningful to the participant may break from suppression faster [6]. This difference in suppression time between stimuli can be used to infer the differences in non-conscious processing before they reach awareness [10]. This breaking-from-suppression (CFS-b) paradigm has been utilized to study processing differences of high-level features including emotions. For facial expressions of emotion, studies using fearful faces provided mixed results [11–15]. Three of them showed that fearful faces tend to break from suppression faster than other emotional facial expressions [11–13], underscoring the attention-grabbing status of fearful facial expressions that was found in masking studies [16].

The current human emotion theories may be incomplete due to an emphasis on the facial expressions; an important question is whether the effects obtained with facial expressions also generalize to bodily expressions of emotions. Bodily expressions are potent emotional signals when perceived consciously [17], as task-irrelevant information under attention manipulation [18], but also do so when processed outside visual awareness. This has been demonstrated in some patients with cortical blindness due to visual cortex lesion, who show affective blindsight, the ability to still be able to process visual emotional signals [19]. For example, emotional bodies presented in the blind visual field elicited activations in subcortical structures including superior colliculus and pulvinar [20], and elicited similar facial muscle reactions to the ones presented in the sighted visual field [21]. Studies with neglect patients also showed that exposure to images with emotional bodies substantially reduced the attentional deficits [22, 23]. Together with masking studies in healthy participants [24, 25], these studies provide evidence for the processing of bodily expressions of emotion outside awareness. In view of the fact that masking methods are not always conclusive [26], convergent evidence from other approaches is desirable.

The CFS paradigm has so far not been used to investigate non-conscious perception of emotions expressed by body postures. In the current study, we used the CFS-b paradigm to examine suppression time differences among stimuli with neutral, fearful, and angry emotions, conveyed by either the face or the body. Our study aimed to answer three questions. First, can CFS also be used to investigate non-conscious emotional body processing and if so, are there differences in suppression times of the different emotions? Second, is the pattern of suppression time differences between emotions similar for bodily and facial expressions? Finally, in view of a debate on this issue in the literature, can we add new evidence about the suppression times under CFS for emotional facial expressions?

## Methods

### Participants

Participants were recruited from the Maastricht University campus (n = 32, mean age = 21.7, ranging from 18–30, with seven male and three left-handed participants). All participants had normal stereo and color vision, normal or corrected-to-normal visual acuity, and no history of neurological disorders. They provided written consent and received either credit points or monetary reward afterwards. The procedure for the study was approved by the Ethics Committee of Maastricht University, and was carried out in accordance with the standards established by the Declaration of Helsinki.

### Stimuli

**Fig 1:**
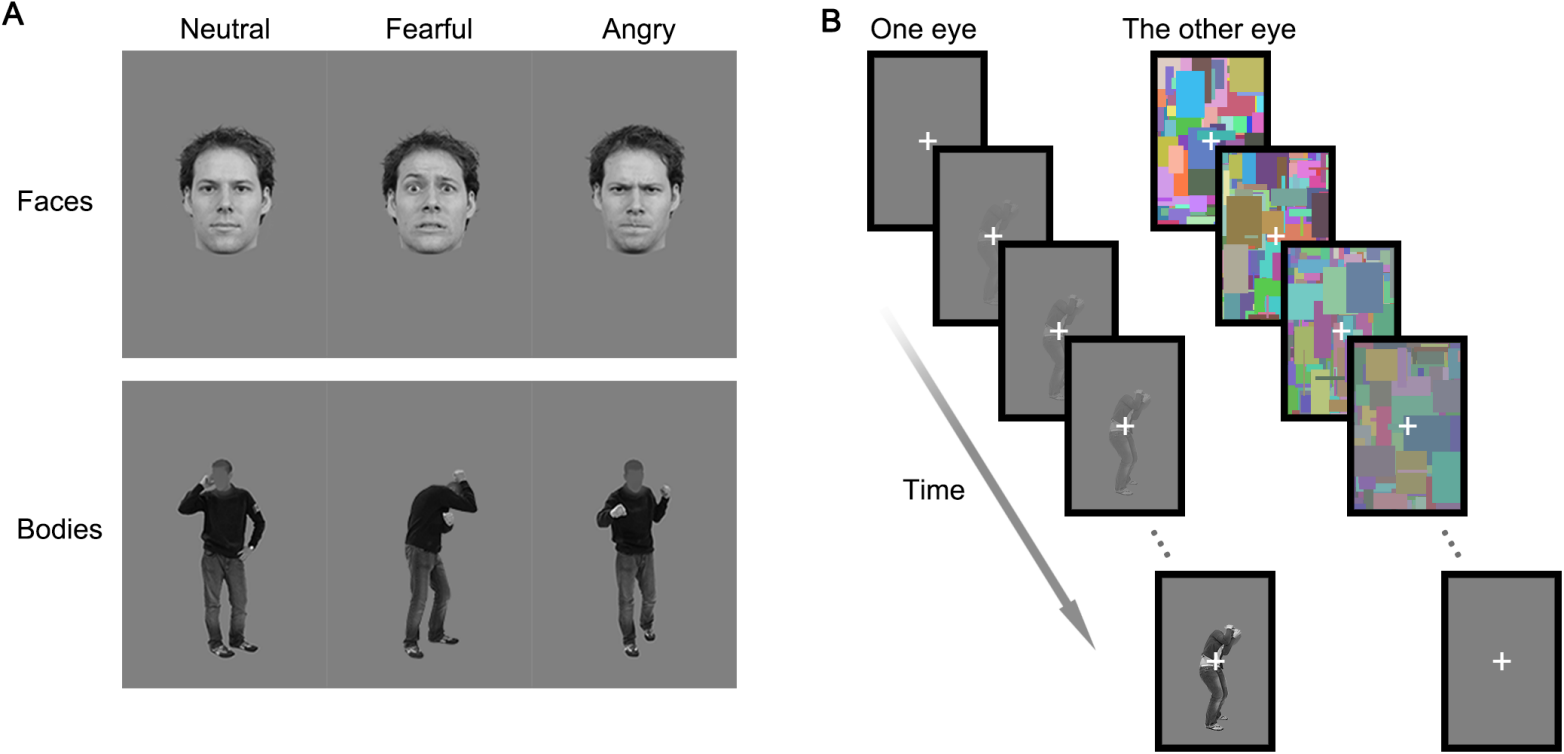
Examples of stimuli and the CFS-b procedure. (**A)** Examples of face and body stimuli, with neutral, fearful and angry emotional facial and bodily expressions were used. (**B)** The CFS-b procedure.

The stimuli were face and body images in grayscale. Each stimulus category had 24 identities, and each identity had a neutral, angry, and fearful expression. The face stimuli were adapted from the Radboud Faces Database [27]. They were aligned at the eye level, and an ellipse (from below the chin to the top of the head, 1.91°×2.67° of visual angle) masked out the visual details for each facial stimulus outside that region. The body stimuli were adapted from Stienen and de Gelder [24], with facial information removed, and aligned at the shoe-level. Neutral stimuli consisted of individuals talking on the phone. The body stimuli spanned within 1.8°×4.3° visual angles. Since our interest lies more on the effects of different emotions within the same stimulus category (either face or body), and the emotions performed by the same individual were photographed under the same lighting conditions and camera settings, to keep the ecological validity, we used the full-contrast images without further balancing the luminance and contrast across emotions, or across stimulus category. Instead, the low-level properties were controlled by performing mixed-models analyses. Fig 1A shows examples of the stimuli used.

### Procedure

The stimuli were presented in MATLAB (the MathWorks, Natick, MA, USA) with Psychtoolbox [28, 29], on an LCD screen (Acer VG248, 3D capable, resolution = 1920×1080, refresh rate = 60 Hz), in a room with dim light. The background of the screen was set to gray (RGB value = 128,128,128). In the middle of the screen, two rectangles (240x160 pixels, 6.4°x4.27° of visual angles) were placed side by side and the centers of the rectangles were 250 pixels apart (visual angle = 6.67°). A frame of 10 pixels delineated the border of the rectangles, and a black fixation cross was placed in the center of each rectangle. The fixation crosses and the frames helped to maintain convergence of the two rectangles for participants.

A chin rest was placed 59 cm away from the screen. Participants viewed the screen through a pair of diopter glasses (the diopter for each lens = 7). The diopters were calculated and chosen according to the visual angle between the two rectangles [30], which would shift each rectangle back to the center of screen upon viewing. A cardboard divider was placed between the screen and the chin rest, dividing the screen into two equal halves. Participants were instructed to fixate on the cross and free-fuse the two rectangles into one. The experiment would start only after participants reported that they could clearly see only one rectangle, and that the view was stable. All participants reported successful and stable fusion. With this setup, either eye of the participant could see only the content of the ipsilateral rectangle.

In a trial, a dynamic noise (160×240 pixels, flashing at 10 Hz) was projected into one of the rectangular frames. The noise images consisted of overlapping and colorful small rectangles (with height and width within 2°). The noise images in each trial were drawn randomly from 600 unique noise images. One stimulus imbedded in the same gray background was projected into the other rectangular frame, as the target stimulus. In each trial, the initial fixation cross changed to white one second before the start of the trial, and remained white throughout the trial. In the inter-trial intervals (length = 4 s), the fixation cross changed back to black. Each trial had a duration of 9 s. The stimulus was gradually ramped up from 0% contrast to full contrast in the first 8 s, and remained at full contrast for another 1 s. The contrast of the noise was ramped down in the first 8 seconds to 0%. Thus in the 9th second only the stimulus was presented in one of the rectangles. Participants were instructed to fixate continuously, to blink as little as possible during a trial, and to press the space bar on the keyboard as soon as they perceive anything else other than the dynamic noise. Fig 1B presents the CFS-b procedure. We did not provide the participants with any prior knowledge about the nature of the suppressed stimuli in order to exclude possible top-down effects.

The face and body stimuli were presented in two separate blocks. The order of the blocks was counterbalanced between participants. Each individual stimulus was presented once in each eye. One block consisted of 144 trials, randomized within the block, and balanced between the eyes. In the middle of each block there was a short break.

After the experiment, participants were asked to free-recall the emotion categories that they recognized, and whether they clearly perceive the whole stimuli at the time they pressed the key. If they could not label the emotion of the body, they were asked to describe the posture.

### Main analyses

The data were analyzed in SPSS and R (R Core Team, Vienna, Austria). For removing outliers, the single-trial suppression times were z-transformed for the data of each block separately, and also across the two blocks for each participant. The data points that exceed four standard deviations (SD) in either one of the z-transformations performed were excluded as outliers. Trials without a response (suppression time ≥ 9 s) were also excluded. In total, 1.13% of the trials were excluded. In the face block, mean number of excluded trials was 1 out of 144 trials (*SD* = 1.29). In the body block, for one participant 28 trials were removed due to no response, while for the other participants mean number of excluded trials was 1 out of 144 trials, *SD* = 1.8. There was no difference between the face and body blocks in the number of outliers removed, *t*(31) = -0.351, *p* = .728.

The raw suppression time (in seconds) was aggregated by participant. A two-way factorial repeated-measure ANOVA was performed, with category (face, body) and emotion (neutral, fear, anger) as within-subjects factors. Paired samples t tests were used for post-hoc testing.

### Mixed models control analyses

We also took low-level properties of the stimuli into consideration by performing mixed models analyses. Since we expect that the low-level properties will affect the two stimuli categories differently, the mixed models analyses were performed separately on the data of the face and the body blocks. As low-level properties, we counted the number of pixels for each body and face stimulus, and obtained the average pixel value for each. We also obtained the root-mean-square contrasts of the whole body stimuli, and of the eye-eyebrow region of the face stimuli.

The pixel count, mean pixel value, and the RMS contrasts were centered, and residualized on the emotion types using SPSS, and the residuals corresponded to the part not explained by the emotion types. The multicollinearity checks were performed for the face and the body data separately. The emotion type, with the residualized pixel number, mean pixel value, and RMS contrast were entered into a backward regression model as predictors. The suppression time was defined as the dependent variable. Multicollinearity checks showed a variance inflation factor below 1.5 for all the predictors, suggesting that they were free of multicollinearity problems. In the following paragraphs the “residualized” will be omitted for conciseness.

The mixed models analyses were performed in R (R Core Team, Vienna, Austria). The linear mixed models were constructed and compared with the lme4 toolbox [31]. For the models, fixed effect predictors included: *emotion type*, *pixel count*, *mean pixel value*, *RMS contrast*; random effects included: *participant*, *stimulus*, where the *stimulus* predictor is a by-item predictor for the stimuli. The details of model comparison steps are described in the result session. The least-squared mean estimates for the fixed effect *emotion type* was obtained using the lsmeans [32] and pbkrtest [33] toolboxes. The models that differed in random effect predictors were fitted by the restricted maximum likelihood method (REML), and the models that differed in fixed effect predictors were fitted by the maximum likelihood method (ML). The final model was then fitted with REML to obtain the linear estimates.

## Results

### Results of the main analyses

There was a significant main effects of category, *F*(1,31) = 71.20, *p* < .000001, η_p_^2^ = 0.70, and of emotion, *F*(2,62) = 8.47, *p* = .0006, η_p_^2^ = 0.21. Importantly, there was a significant interaction between category and emotion, *F*(2,62) = 28.64, *p* < .001, η_p_^2^ = 0.48.

Post-hoc paired-samples t-tests revealed that the suppression time for angry faces (*M* = 3.45, *SE* = 0.21) was significantly longer than either neutral (*M* = 3.23, *SE* = 0.19), *t*(31) = 3.93, *p* < .001, *r* = 0.57, or fearful faces (*M* = 3.23, *SE* = 0.18), *t*(31) = 3.60, *p* = .001, *r* = 0.54. The difference between neutral and fearful facial expressions was not significant, *t*(31) = 0.04, *p* = .97, *r* = 0.01.

**Fig 2:**
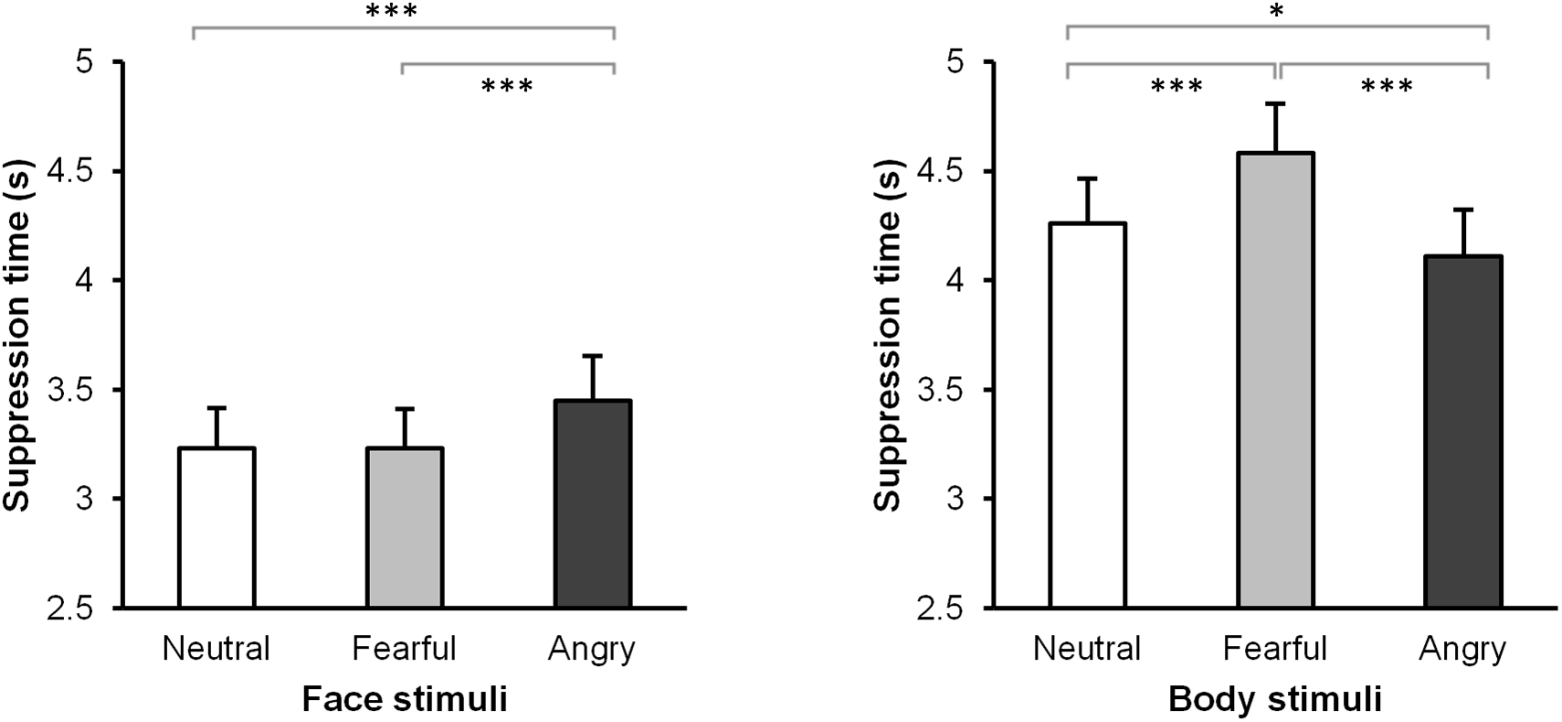
Results of the face and body blocks. Suppression time for neutral, fearful, and angry facial and bodily expressions. Error bars represent standard errors. *: p < .05; ***: p < .001.

In contrast, suppression time for angry bodies was significantly shorter (*M* = 4.11, *SE* = 0.21) compared to fearful (*M* = 4.58, *SE* = 0.23), and neutral (*M* = 4.26, *SE* = 0.20) bodies, with *t*(31) = 7.67, *p* < .001, *r* = 0.81 and *t*(31) = 2.63, *p* = .013, *r* = 0.43, respectively. Suppression time for fearful bodies was longer compared to neutral bodies, *t*(31) = -4.29, *p* < .001, *r* = 0.61 (Fig 2).

To test if the order of the blocks influenced the results, we added order (face→body versus body→face) as a between-subjects factor in the repeated-measure ANOVA. There was no main effect of order, *F*(1,30) = 1.09, *p* = .31, η_p_^2^ = 0.04, or emotion by order interaction, *F*(2,60) = 0.98, *p* = .38, η_p_^2^ = 0.03. A significant interaction between category and order appeared, *F*(1,30) = 35.34, *p* = .000002, η_p_^2^ = 0.54. The group with the body block following the face block (*n* = 16, *M* = 3.87, *SE* = 0.28) showed shorter suppression times for the body category compared to the group with the face block following the body block (*n* = 16, *M* = 4.76, *SE* = 0.28), *t*(30) = -2.23, *p* = .03, *r* = 0.38. This was not the case for faces, *t*(30) = 0.24, *p* = .81, *r* = 0.04 (face→body: *M* = 3.35, *SE* = 0.29, and body→face, *M* = 3.26, *SE* = 0.25). Crucially, the three-way interaction between order, emotion and category was not significant, *F*(2,60) = 0.47, *p* = .63, η_p_^2^ = 0.02. Order did not influence the main results.

In addition, we checked whether the participants based their decision on a consistent basis within each block, and if they didn’t wait too long for a clear perception of the stimuli. All participants reported that they pressed the response button as soon as possible, but their partial-whole perception varied when pressing the button: 20 participants saw the whole stimuli most of the time, 7 participants saw partial features most of the time, and 5 participants had a mixed perception. To test if seeing the whole or partial of the stimuli affected the observed results, we added it as a between-subject factor in the repeated-measure ANOVA. There was no main effect of stimuli completeness, *F*(2,29) = 0.27, *p* = .77, η_p_^2^ = 0.02. No interaction of stimuli completeness was present with either category, *F*(2,29) = 0.51, *p* = .60, η_p_^2^ = 0.03, or emotion, *F*(2,29) = 1.57, *p* = .19, η_p_^2^ = 0.10, or with category and emotion together, *F*(4,58) = 0.94, *p* = .45, η_p_^2^ = 0.06.

### Results of the mixed models control analyses

**Face stimuli.** First, model 1 was constructed with one fixed effect predictor (*emotion type*), and two separate random effect predictors (*participant*, *stimulus*). An intercept and random slope for *emotion type* was included in the *participant* predictor. The model 1 had the maximal random effect structure justified by the data. A second model (model 2) excluded the random slope of *emotion type* in the *participant* predictor. Comparison of these two models showed that model 1, with the random slope, better described the data, *χ* ^*2*^(5) = 13.86, *p* = .016. The random slope was kept in the subsequent models.

Model 3, 4 and 5 were constructed by adding a second fixed effect predictor, respectively: *pixel count* (model 3), *mean pixel value* (model 4), *RMS contrast* for the eye-eyebrow region (model 5). Model 5 with *RMS contrast* described the data better than model 1, *χ* ^*2*^(1) = 24.52, *p* < .001. The estimate (*β*) for the predictor RMS contrast was -0.03, *SE* = 0.005, *t* = -5.41, *p* = .0001. The *p* value was obtained using the pbkrtest toolbox, with 10000 simulation samples. Subsequently adding *pixel count* and *mean pixel value* to model 5 respectively wasn’t justified by the *χ* ^*2*^ change. The model 5 was selected as the final model.

**Fig 3:**
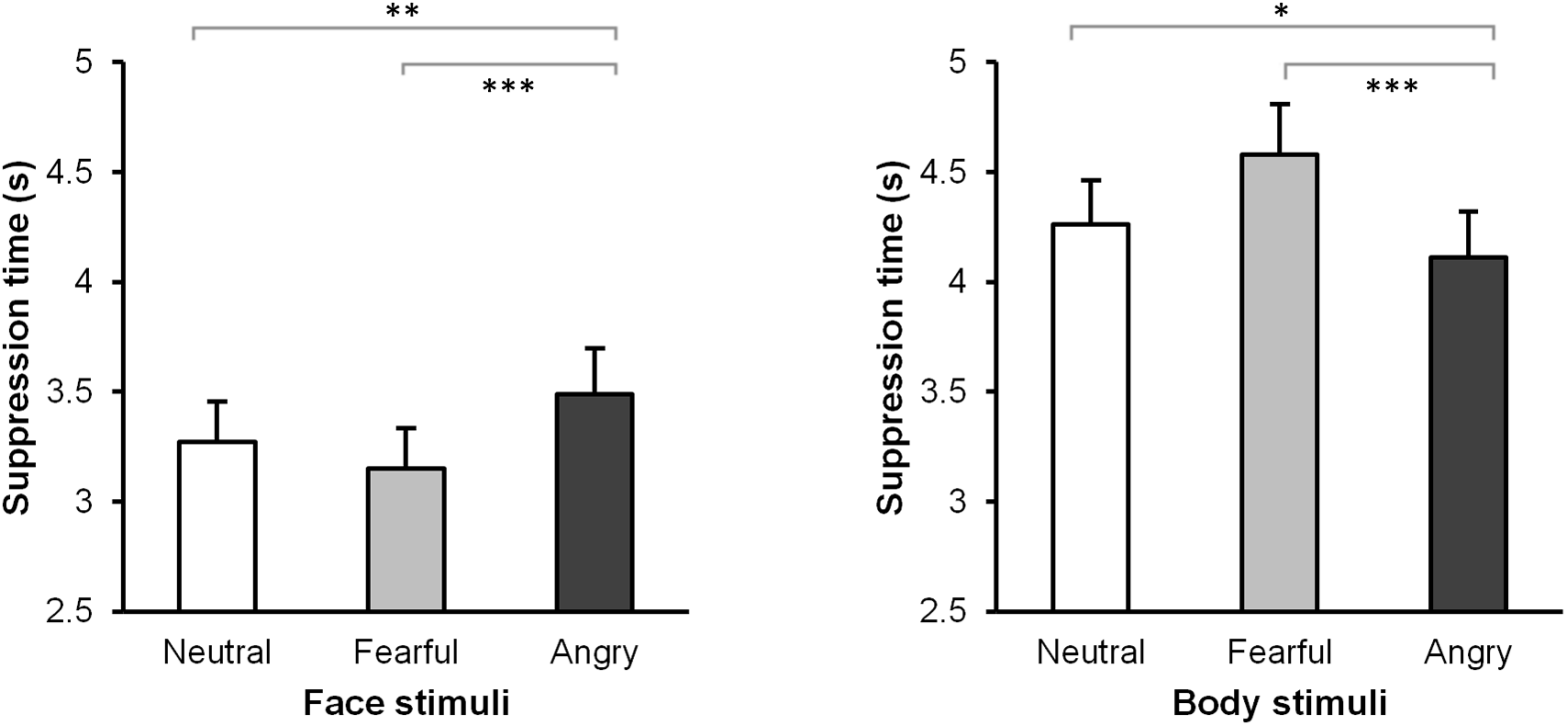
Suppression time for neutral, fearful, and angry facial and bodily expressions after correcting for low-level features unrelated to emotion. Error bars represent standard errors. *: *p* < .05; **: *p* < .01; ***: *p* < .001.

In the final model, with *emotion type* and *RMS contrast* as fixed effect predictors, the least squared means of the contrasts also showed similar results to those in the ANOVA analysis, neutral versus fearful faces: *t*(38.93) = 1.88, *p* = .159; neutral versus angry faces: *t*(32.42) = -3.73, *p* = .002, fearful versus angry faces: *t*(39.40) = -4.99, *p* < .0001.The estimate for fearful faces was 0.12 s shorter than neutral faces, which was a pattern also shown in the literature, but this difference was still not significant (Fig 3 and Table 1).

**Table 1:** Estimates (*β* values, in seconds), standard errors, and *t* values for the fixed-effect predictors in the analyses for the face and body blocks.

Predictor	Estimate (*s*)	SE	*t*
Face stimuli			
Intercept (neutral faces)	3.275	0.184	17.845
Fearful vs. neutral faces	-0.120	0.062	-1.923
Angry vs. neutral faces	0.216	0.056	3.834
RMS Contrast of the eye region	-0.031	0.006	-5.411
Body stimuli			
Intercept (neutral bodies)	4.264	0.202	21.099
Fearful vs. neutral bodies	0.317	0.083	3.846
Angry vs. neutral bodies	0.152	0.066	-2.308
Mean pixel value of the stimulus	0.009	0.001	6.708

The *p* values in the analyses were obtained by the lsmeans and pbkrtest toolboxes, thus were reported separately in the text.

### Body stimuli

The model comparisons for the body stimuli followed a similar procedure as those for the face stimuli. Model 1 and 2 were similarly constructed, with a random slope for *emotion type* in the *participant* predictor, either included (model 1) or omitted (model 2). Model 1 better described the data, *χ* ^*2*^(5) = 14.76, *p* = .01. The random effect structure was kept constant in the subsequent models.

In models 3, 4 and 5, comparing to model 1, *pixel count* (model 3), *mean pixel value* (model 4), *RMS contrast* (model 5) were added as the second fixed effect predictor, respectively. Comparisons showed that in the 3 models, model 4 with the *mean pixel value* for the whole body stimulus described the data significantly better than model 1, *χ* ^*2*^(0) = 34.84, *p* < .001. The estimate (*β*) for the predictor *mean pixel value* was 0.009, SE = 0.001, *t* = 6.58, *p* = .0001 (The *p* value was obtained by comparing model 5 with model 1, using a parametric bootstrapping model comparison test in the pbkrtest toolbox, with 10000 simulation samples). Subsequently, adding *pixel count* and *RMS contrast* to model 4 respectively, was not justified by the *χ* ^*2*^ change. The model 4 was selected as the final model.

In the final model, with *emotion type* and *mean pixel value* as fixed effect predictors, the least squared means of the contrasts showed a similar pattern for the emotion as in the ANOVA analysis: neutral versus fearful bodies: *t*(41.69) = -3.76, *p* = .0015; neutral versus angry bodies: *t*(38.57) = 2.26, *p* = .073, fearful versus angry bodies: *t*(40.28) = 6.51, *p* < .0001 (Fig 3 and Table 1).

## Discussion

Our goal was threefold: 1) to use the same CFS-b paradigm to investigate perception of bodily expressions without visual awareness, 2) to investigate the possible differences in pattern of suppression time differences among emotions for face and body stimuli, and 3) to add to the available evidence on the perception of facial expressions using the CFS-b paradigm.

First, we showed that the suppression times did differ among bodily expressions. Specifically, angry bodies broke suppression faster, while fearful bodies broke suppression slower. Compared to fearful bodies, angry bodies represent a much more direct threat to the observer, which tend to trigger avoidance and escape behaviors [34]. Consistent with this notion, previous fMRI studies showed increased activity in the anterior temporal lobe, the premotor cortex, and the ventromedial prefrontal cortex, suggesting automatic defense-related action preparation, in response to angry bodies [34, 35]. This involvement of the action-preparation network might be the mechanism that facilitated the breaking from suppression for angry bodies in our study. The posterior superior temporal sulcus might also play a role, as applying transcranial magnetic stimulation to this region facilitated the detection of changes in the masked angry bodies but not neutral bodies [36].

For facial expressions, our results showed that angry faces broke from suppression slower than neutral and fearful faces. This is in accordance with the study of Gray et al. [12], showing that angry faces broke from suppression slower than neutral faces. Our result did not show a significant difference between fearful and neutral faces. While several CFS-b studies with fearful faces showed a shorter suppression time than neutral faces [11–13], two other studies showed a non-significant difference in their healthy controls [14, 15]. This discrepancy might be caused by the large number of identities (24 identities) in the facial stimuli used in the current study, compared to the number (4 or 8 identities) in the aforementioned studies. The large number of stimulus identities here introduced ecological validity, but may also have introduced variability in the responses of the participants. In general, the pattern of suppression time among the three facial expressions shown in our results does not depart from that in the literature. Further research is needed to better understand the role of individual differences in conscious and non-conscious fear perception. For example, individual differences in sensitivity have been shown for fearful [37] and angry expressions [25].

Finally, our results also provide evidence, regarding the suppression time patterns among emotions and possible differences between facial and bodily expressions. Our results already showed a general suppression time difference between faces and bodies, with bodies being suppressed longer. This is consistent with the literature [38], and indicated a difference in non-conscious processing of faces and bodies. The difference in the pattern obtained for face and bodily expressions may therefore indicate that a degree of category specificity may be present at the level of non-conscious emotion signal processing.

Notwithstanding the fact that face and bodily expressions may convey the same meaning, they are actually very different visual stimuli once one gets away from asking subjects for explicit cognitive recognition of the emotion. At the level of visual features, faces and bodies clearly convey the emotional information by very different means. The facial emotion is conveyed by the fine details of internal facial features, including the salient eye-eyebrow region, which is thought to be driving the detection of faces under CFS [11, 12]. There is evidence pointing to eye-specific mechanisms for face perception, in which the amygdala may play an important role: eye contact alone activated the amygdala for a complete cortical blind patient [39], single-cell recordings in monkey amygdala also found specialized cells for eye contact [40]. The bodily emotion expression is rather conveyed by the position and movements of the body parts leading to better expression recognition at a further distance.

From the vantage point of basic emotion theory, it has been shown that the same brain areas are being activated by the same emotion, whether the stimuli are faces, bodies or voices [41]. However, it is not clear so far whether the categorical structure and the representation of the emotion labels is the same at earlier processing levels, especially at a non-conscious level [42, 43]. Studies comparing the neural basis showed that emotional face and body processing involved overlapping brain regions, including the fusiform gyrus, superior temporal gyrus, and the middle occipital gyrus; however the calcarine sulcus, cerebellum, superior frontal gyrus and anterior cingulate gyrus were more involved in face processing, while the superior occipital gyrus, parieto-occipital sulcus and the intraparietal sulcus were more involved in body processing [44].

These processing differences may indicate stimulus and emotion selective interactions with the parietal attention network [45] for face and body stimuli, thus contributing to different patterns of faces and bodies in reaching awareness. For bodily expressions, studies showed that activation increases in the motor system [46]. Bodily expressions may trigger the involvement of more action-perception and -preparation processes [17, 36, 47, 48]. This may be an important part of the mechanism underlying the shorter suppression time for angry bodies in our study.

A complementary explanation may be provided by processes related to proprioception. A recent CFS study showed that when participants saw a suppressed hand image in the same pose as their actual hand, the suppressed hand broke suppression faster [49]. This might indicate that the matching of the proprioceptive information and the unconsciously observed visual information may have a facilitatory effect for stimuli to reach awareness [49]. Another recent study comparing neural correlates of seen and unseen bodily expressions argues in favor of the role of the insula and sensorimotor processes in the transition from non-conscious perception to awareness [23]. The results indicated that the integration between the mapping of bodily changes at the neural level and the peripheral arousal is critical for the conscious visual experience of emotional signals [23].

Apart from the category-specific differences between the stimuli, the depth of suppression with the CFS paradigm is related to the spatial and temporal properties of both the stimuli and the masks, such as contrasts [50]. Our research questions mainly focused on the different patterns elicited by the same emotions in faces and bodies, rather than the absolute suppression time between the two categories. The spatial-temporal effects of the stimuli and the masks are compatible within each category. Still, could the present results be explained by these low level properties of the stimuli, and could this factor thus, at least indirectly, be responsible for the observed asymmetric patterns between faces and bodies? For face stimuli, three studies showed that fearful faces broke from suppression faster than neutral faces [11–13]. Two of them suggested that faster access was caused mainly by low-level features of the eye and eyebrow region (e.g. the local high contrast in the region), but not by the emotional information [11, 12]. Here we used a large number of different identities in our stimuli (24 identities for both face and body stimuli), introducing variability in the lower-level visual properties. To further control for the effect of the low-level features of the stimuli, we did an analysis that residualized the low-level features on the emotion types, and separated the effect of a low-level feature into two parts: one part that is systematically introduced by the stereotypical features relating to specific emotion types (such as the lowered RMS contrast in the eye-eyebrow region for fearful faces, caused by the larger area of eye whites, which is related to possible eye-specific mechanisms), and a second part that is pure low-level variances (the residuals, which might include the influence of the facial skin tone variation across individuals that is not related to the emotion). We examined whether this second part would be important in affecting the suppression time. For both the body and face stimuli, the suppression time was indeed influenced by the lower-level visual properties of the stimuli, but only to a very small extent. For the face and body data respectively, the RMS contrast of the eye-eyebrow region and the mean pixel value for the whole stimulus described the data well, but the estimate was one to two orders of magnitude smaller than that for the emotion type. These results indicated that in the current study, the variance of the low-level feature unrelated to the emotions was not necessarily the dominant factor for the effect observed in CFS experiments. The difference between the emotion contents, which was processed at a higher level, was still the biggest factor for the difference in suppression times.

## Conclusion

We examined the suppression time under CFS for neutral, fearful and angry facial and bodily expressions. Our results showed that fearful and angry bodily expressions differ in suppression time, with angry bodies breaking from suppression faster, suggesting a different involvement of the action perception network. In contrast, angry faces broke from suppression more slowly comparing to the other two facial expressions. These results indicate different mechanisms and neural networks for non-conscious perception of emotional faces and bodies. Future fMRI studies are needed to further explain the processing mechanisms for these emotions under non-conscious viewing conditions.

## Supporting Information

S1 Data Data of the CFS-b experiment. (XLSX)
